# Editorial: Characteristics and behaviors of hematopoietic stem cells and leukemia stem cells in hematologic malignancies

**DOI:** 10.3389/fonc.2022.1037202

**Published:** 2022-10-27

**Authors:** Haojian Zhang, Pengxu Qian

**Affiliations:** ^1^ The State Key Laboratory Breeding Base of Basic Science of Stomatology & Key Laboratory of Oral Biomedicine Ministry of Education, School & Hospital of Stomatology, Medical Research Institute, Wuhan University, Wuhan, China; ^2^ Frontier Science Center for Immunology and Metabolism, Medical Research Institute, Wuhan University, Wuhan, China; ^3^ Center of Stem Cell and Regenerative Medicine, and Bone Marrow Transplantation Center of the First Affiliated Hospital, Zhejiang University School of Medicine, Hangzhou, China; ^4^ Liangzhu Laboratory, Zhejiang University Medical Center, Hangzhou, China; ^5^ Institute of Hematology, Zhejiang University & Zhejiang Engineering Laboratory for Stem Cell and Immunotherapy, Hangzhou, China

**Keywords:** leukemia stem cell, hematopoietic stem cell, hematopoietic malignancies, chronic myeloid leukemia, acute myeloid leukemia, drug resistance

Hematopoietic homeostasis is tightly controlled by complex molecular networks through the whole lifetime. Genetic and epigenetic alterations occurred in hematopoietic stem and progenitor cells (HSPCs) impair these networks and disrupt the balance of hematopoietic homeostasis, and subsequently transform HSPCs into leukemia stem cells (LSCs) resulting in the development of hematologic diseases, such as myeloid leukemia, and myeloproliferative neoplasms. Thus, although LSCs share many similar biological features with normal HSPCs. However, unique characterizations and behaviors of LSCs are gradually appreciated. Moreover, due to the biological features of LSCs and the limited ability of current chemotherapy regimens in targeting LSCs, it is widely accepted that the existence of LSCs largely contributes to the relapse of hematological malignancies in clinic. Therefore, targeting LSC population is an an essential prerequisite for curing hematologic diseases including leukemias. Hematopoietic stem cells (HSCs) prefer to reside in a quiescent state, which is achieved by multiple strategies, including lower protein synthesis, glycolysis, and unique stress-responsive system. Along with the transformation of LSCs, a series of programs, e.g. epigenetics and metabolism, occur adaptive changes. Therefore, in order to be successful in targeting LSCs, it is necessary to fully understand the biology of LSCs, and to decipher the differences between normal HSCs and LSCs. This Research Topic focuses on recent advances in understanding the differences between normal HSCs and LSCs, as well as the underlying mechanisms for drug resistance of LSCs, which enable us to develop new strategies for targeting LSCs.

Acute myeloid leukemia (AML) is one of the most fatal hematopoietic stem/progenitor cell diseases, and exhibits great heterogeneity as patients with AML frequently carry many cytogenetic and molecular alterations. Currenttly, AML treatments often fail to target leukemic stem cells, and relapse and refractory of this disease remain big challenges in clinic. Various intrinsic and extrinsic factors influence drug sensitivity of LSCs and leukemia relapse. Niu et al. provide a comprehensive review about the underlying mechanisms for primary resistance and chemotherapy-related remolding of LSCs. They focus on the following aspects: the inherent dormant state of LSCs, enhanced expression of ATP-binding cassettes transporters, apoptotic alterations, senescence-related mechanisms, adaptive metabolic reprogramming, epigenetic alterations, bone marrow niches. Thus, the work delineates a landscape of how LSCs behave. Metabolic reprogramming is appreciated as one of cancer hallmarks, which might be the Achilles’ heel of cancer stem cells. Increasing evidence reveal the unique metabolic characteristic of LSCs. In this Research Topic, Peng et al. particularly focus on metabolic alterations of LSCs, and compile an interesting review article entitled “Targeting mitochondrial oxidative phosphorylation eradicates acute myeloid leukemia stem cells”. They summarize the most recent advances on the regulatory mechanims of the electron transport chain (ETC) and tricarboxylic acid (TCA) cycle in mitochondrial oxidative phosphorylation, and discuss potential therapeutic strategies for targeting LSCs. Taken together, these two excellent reviews enable us to understand the molecular mechanisms of LSC drug resistance, and may provide a better view and direction for further investigating LSC characteristics and behaviors in the future.

Chronic myeloid leukemia (CML) is a clonal myeloproliferative disease that originates from abnormal HSCs harboring the Philadelphia (Ph) chromosome, that is, a reciprocal translocation between chromosome 9 and 22 [t (9; 22) (q34; q11)]. This genetic chromosome translocation cause the formation of the chimeric BCR-ABL oncoprotein that acts as tyrosine kinase with a constitutive activity. Tyrosine kinase inhibitors (TKI), such as imatinib, have revolutionized the therapy strategy for CML treatment in clinic, and been very successful in improving the survival of patients with CML. Unfortunately, treatment-free remission after discontinuing TKIs becomes a new goal for CML treatment. In this topic, Chen et al. summarize recent studies on TKI discontinuation, and discuss the factors (e.g. clinical indicator, immunological indicator, and others) for predicting CML recurrence at the molecular level, the monitoring methods of LSCs, and potential strategies for killing LSCs in CML. Overall, this work provides us an insight and a hope for reaching treatment-free remission of patients with CML.

There are a lot of challenges for acute myeloid leukemia treatment, and chemotherapy is still the first-line treatment for acute myeloid leukemia. While allogeneic hematopoietic stem cell transplantation (allo-HSCT) is thought to be a best way to cure AML, it is also associated with transplant-related morbidity and mortality. Infectious complication is one of the important reasons for the morbidity and mortality of patients undergoing allo-HSCT. Therefore, a better program for infection prevention and management is critical for improving allo-HSCT. In here, Malagola et al. share an innovative program called BATMO (Best-Antimicrobial-Therapy-TMO) for infection prevention and management used in their center since 2019. This study suggests that the BATMO program is safe and obviously prevents patients from Clostridiodes difficile infections and CMV reactivations. In summary, this work provides an example for managing antimicrobial therapy to prevent infection issue for patients with allo-HSCT.

Together, the collection of studies in this Research Topic provides us keen insights into the underlying mechanisms for drug resistance of LSCs for AML and CML, which might enlighten us to develop new strategies for targeting LSCs. This topic also shares an excellent protocol for infection prevention and management for allo-HSCT.

## Author contributions

HZ wrote the manuscript, and PQ edited it. All authors contributed to the article and approved the submitted version.

## Acknowledgments

We thank all the authors of the publication collected in this Research Topic for their contributions. We also thank all the staff of Frontiers in Oncology who manage and support this Research Topic.

## Conflict of interest

The authors declare that the research was conducted in the absence of any commercial or financial relationships that could be construed as a potential conflict of interest.

## Publisher’s note

All claims expressed in this article are solely those of the authors and do not necessarily represent those of their affiliated organizations, or those of the publisher, the editors and the reviewers. Any product that may be evaluated in this article, or claim that may be made by its manufacturer, is not guaranteed or endorsed by the publisher.

